# Hyperparasitic Fungi on Black Mildews (Meliolales, Ascomycota): Hidden Fungal Diversity in the Tropics

**DOI:** 10.3389/ffunb.2022.885279

**Published:** 2022-05-24

**Authors:** Miguel A. Bermúdez-Cova, Armando J. Cruz-Laufer, Meike Piepenbring

**Affiliations:** ^1^Mycology Research Group, Faculty of Biological Sciences, Goethe University Frankfurt am Main, Frankfurt am Main, Germany; ^2^Departamento de Biología de Organismos, División de Ciencias Biológicas, Universidad Simón Bolívar, Caracas, Venezuela; ^3^Centre for Environmental Sciences, Faculty of Sciences, Hasselt University, Diepenbeek, Belgium

**Keywords:** hyperparasitism, hyperparasitic fungi, Meliolales, checklist, Ascomycota, tritrophic interaction, network analysis, parasitism

## Abstract

Hyperparasitism on plant-parasitic fungi is a widespread but rarely studied phenomenon. Here, for the first time, we compile in a checklist information provided by peer-reviewed literature for fungi growing on colonies of black mildews (Meliolales, Ascomycota), a species-rich group of tropical and subtropical plant-parasitic microfungi. The checklist contains information on 189 species of contact-biotrophic microfungi in 82 genera. They belong to seven morphological groups: dematiaceous hyphomycetes, moniliaceous hyphomycetes, pycnidioid, perithecioid, catathecioid, and apothecioid fungi. By the fact that species accumulation curves do not reach saturation for any tropical country, it is evident that the knowledge of the diversity of hyperparasitic fungi on Meliolales is incomplete. A network analysis of records of hyperparasitic fungi, their host fungi and host plants shows that genera of hyperparasitic fungi are generalists concerning genera of Meliolales. However, most species of hyperparasitic fungi are restricted to meliolalean hosts. In addition to hyperparasitic fungi, diverse further microorganisms use meliolalean colonies as ecological niche. Systematic positions of most species are unknown because DNA sequence data are lacking for species of fungi hyperparasitic on Meliolales. We discuss the specific challenges of obtaining DNA sequence data from hyperparasitic fungi. In order to better understand the diversity, evolution and biology of hyperparasitic fungi, it is necessary to increase sampling efforts and to undertake further morphological, molecular, and ecological studies.

## 1. Introduction

The term hyperparasite refers to an organism that parasitizes another parasitic organism. Hyperparasitism caused by fungi is rather widespread in nature, but it is a phenomenon that has been poorly studied (Haelewaters et al., 2018a, 2021). Several authors have reviewed this type of interaction (Barnett, 1963; Boosalis, 1964; Barnett and Binder, 1973; Cooke, 1977; Hawksworth, 1981; Haelewaters et al., 2018b; Sun et al., 2019). Fungi are able to parasitize parasitic organisms from different kingdoms (Moore et al., 2020). In this review, we consider fungi parasitic on plant-parasitic fungi. For a fungus to be considered a hyperparasite, it needs to impact the host fitness through one or more modifications, otherwise it would be a hypermutualist or hypercommensal (Boosalis, 1964; Northrup et al., 2021).

Biotrophic plant-parasitic microfungi are frequently colonized by hyperparasitic fungi, many of which can penetrate the hyphae, the spores and/or the reproductive structures of their hosts (Gams et al., 2004). Some of these parasites attack specific groups of plant pathogens and are of interest as potential biocontrol agents, such as *Ampelomyces* spp., natural occurring hyperparasites of powdery mildews (Huth et al., 2021). The most common hosts include powdery mildews (Erysiphales), black mildews (Meliolales), rusts (Pucciniales), smuts (Ustilaginales), and Phyllachorales (Hawksworth, 1981; Gams et al., 2004). For the present review, we focus on hyperparasitic fungi on species of Meliolales.

**Figure 1:**
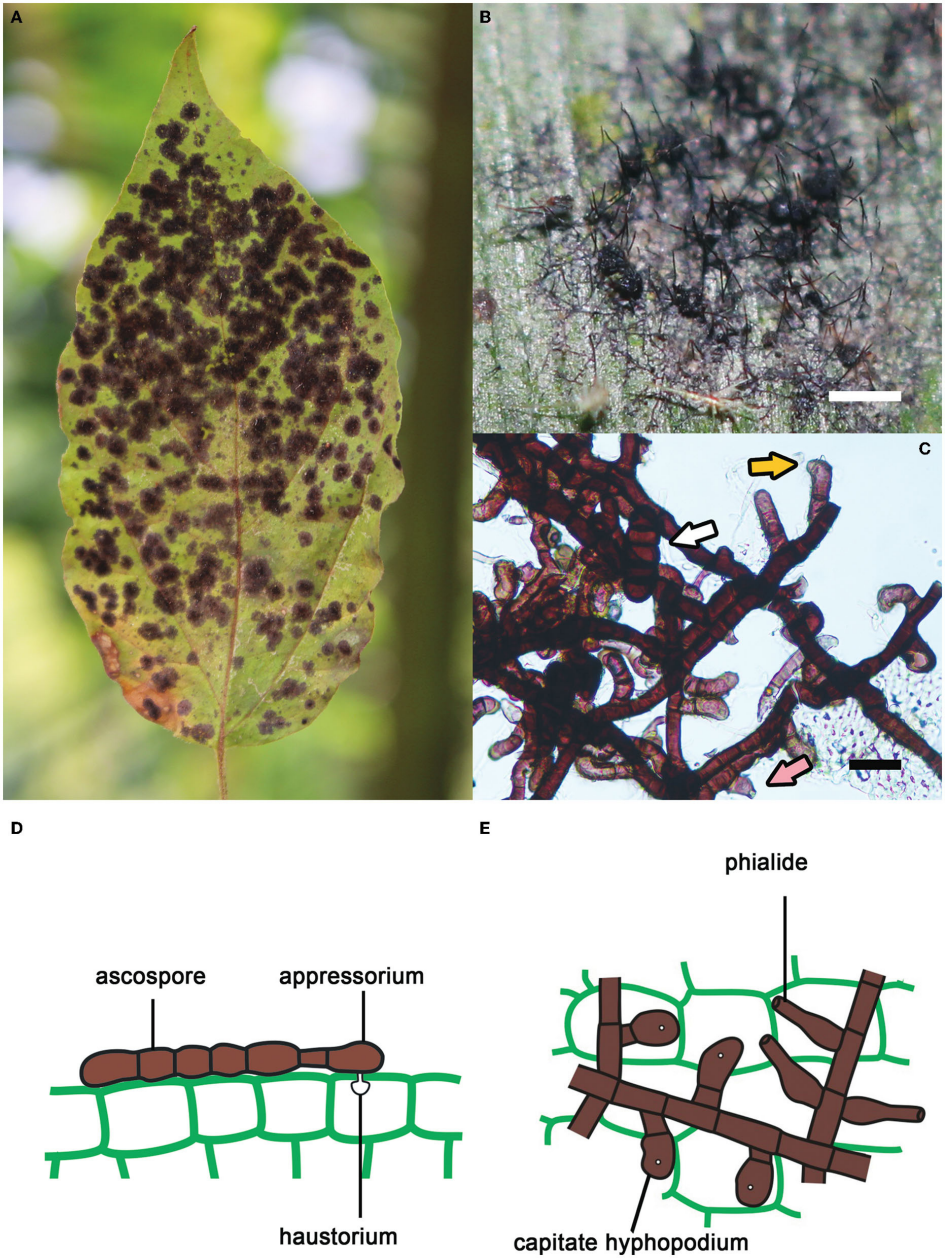
Key features of species of Meliolales. **(A)** Black colonies of *Meliola clerodendricola* on a leaf of *Clerodendrum* sp. **(B)** Superficial hyphae, perithecia and setae of *Meliola* sp. on a leaf of *Olyra latifolia*. Bar, 1 mm. **(C)** Hyphae of *Meliola mangiferae* with capitate hyphopodia (yellow arrow), a phialide (pink arrow) and a septate ascospore (white arrow). Bar, 20 μm. **(D,E)** Schematic drawings of cells of *Meliola* spp. **(D)** Ascospore on the surface of host tissue with a capitate hyphopodium including an appressorium penetrating the wall of the epidermis. **(E)** Hyphae with capitate hyphopodia and phialides.

Meliolales (Sordariomycetes, Ascomycota) form a large order of biotrophic, obligate parasitic fungi in the tropics and subtropics. It comprises 3,064 species, with *Meliola* being the most species-rich genus (1701 spp.; Jayawardena et al., 2020). Species of the order develop on leaves, petioles, twigs and sometimes fruits of vascular plants (Piepenbring et al., 2011; Hongsanan et al., 2015; Zeng et al., 2017). They are known as “black mildews”, as they produce black colonies that are composed of dark, thick-walled, branched, superficial hyphae (Figure 1; Rodriguez Justavino et al., 2015). These hyphae carry numerous short, lateral branches called hyphopodia. Capitate hyphopodia are formed by a foot cell and a globose/lobate terminal cell. This terminal cell acts as an appressorium. A peg formed by the appressorium penetrates the leaf surface and forms a haustorium inside the epidermal host cell to absorb nutrients. Other lateral branches, the phialides, consist of a single, bottle-shaped cell, which can form small spores at the tips. These spores can function as conidia or spermatia, but they have been poorly studied. Meliolalean fungi form perithecia containing asci with dark brown, transversely septate ascospores. Most species present long setae attached to superficial hyphae and/or perithecia (Piepenbring et al., 2011; Piepenbring, 2015).

Infections by species of Meliolales result in a reduction of chlorophyll, starch, sugar, proteins and aminoacids in the affected areas of the plant host (Hosagoudar et al., 1997; Old et al., 2003; Rodriguez Justavino and Piepenbring, 2007). Respiration rates and the temperature of the infected areas may increase due to the lesions and the black color. Photosynthetic activity may be reduced (Hosagoudar et al., 1997; Hongsanan et al., 2014). Heavy infections caused by Meliolales result in a “dirty” appearance of the hosts, thus, reducing their economic value as ornamental plants (Hosagoudar et al., 1997). However, these fungi are not known to cause significant damage to crops (Hosagoudar, 2003).

Hyperparasitic fungi of several genera, mainly belonging to Dothideomycetes or Sordariomycetes, have been reported on species of Meliolales (Deighton and Pirozynski, 1972). These hyperparasites frequently overgrow the entire colonies of the black mildews until the presence of the meliolalean host may be proved only by careful search under a light microscope. Several species of hyperparasitic fungi may be found on the same leaf and even on the same colony (Stevens, 1918; Ciferri, 1955).

Information about fungal hyperparasites on species of Meliolales is scattered throughout literature and no exact number of known species has been reported to date. Most species have been described based on morphology before the widespread use of molecular techniques in fungal taxonomy. Therefore, the modern systematic position of many species of hyperparasitic fungi is unknown. In this review, we compile information available on fungi that parasitize colonies of Meliolales, to highlight knowledge gaps and to aid conceptualization of future research projects.

## 2. Hyperparasitic Fungi on Meliolales: Literature Review

### 2.1. Mode of Host Interaction

Hyperparasitic fungi are classified into two groups based on the mode of parasitism and the effects on the fungal host: necrotrophic and biotrophic parasites (Boosalis, 1964; Barnett and Binder, 1973; Jeffries, 1995; Benjamin et al., 2004; Sun et al., 2019). Necrotrophic hyperparasites invade and kill their fungal hosts, while biotrophic hyperparasites take nutrients from living cells of the fungal host (Jeffries, 1995; Moore et al., 2020). The relationship between the biotrophic parasite and the fungal host is physiologically balanced. The cytoplasm of the host remains functional (Jeffries, 1995). Depending on the type of interaction, i.e., the parasite-host interface, biotrophic hyperparasites are classified into three groups (Barnett and Binder, 1973; Jeffries, 1995; Sun et al., 2019; Moore et al., 2020):

- **Intracellular biotrophs**. Hyphae of the hyperparasite enter the cells of the host fungus.
- **Haustorial biotrophs**. Parts of cells of the hyperparasite penetrate into cells of the host fungus and form haustoria for nutrient uptake.
- **Contact/fusion biotrophs**. Cells of the hyperparasite are in close contact and/or fuse with the cells of the host fungus.

**Figure 2:**
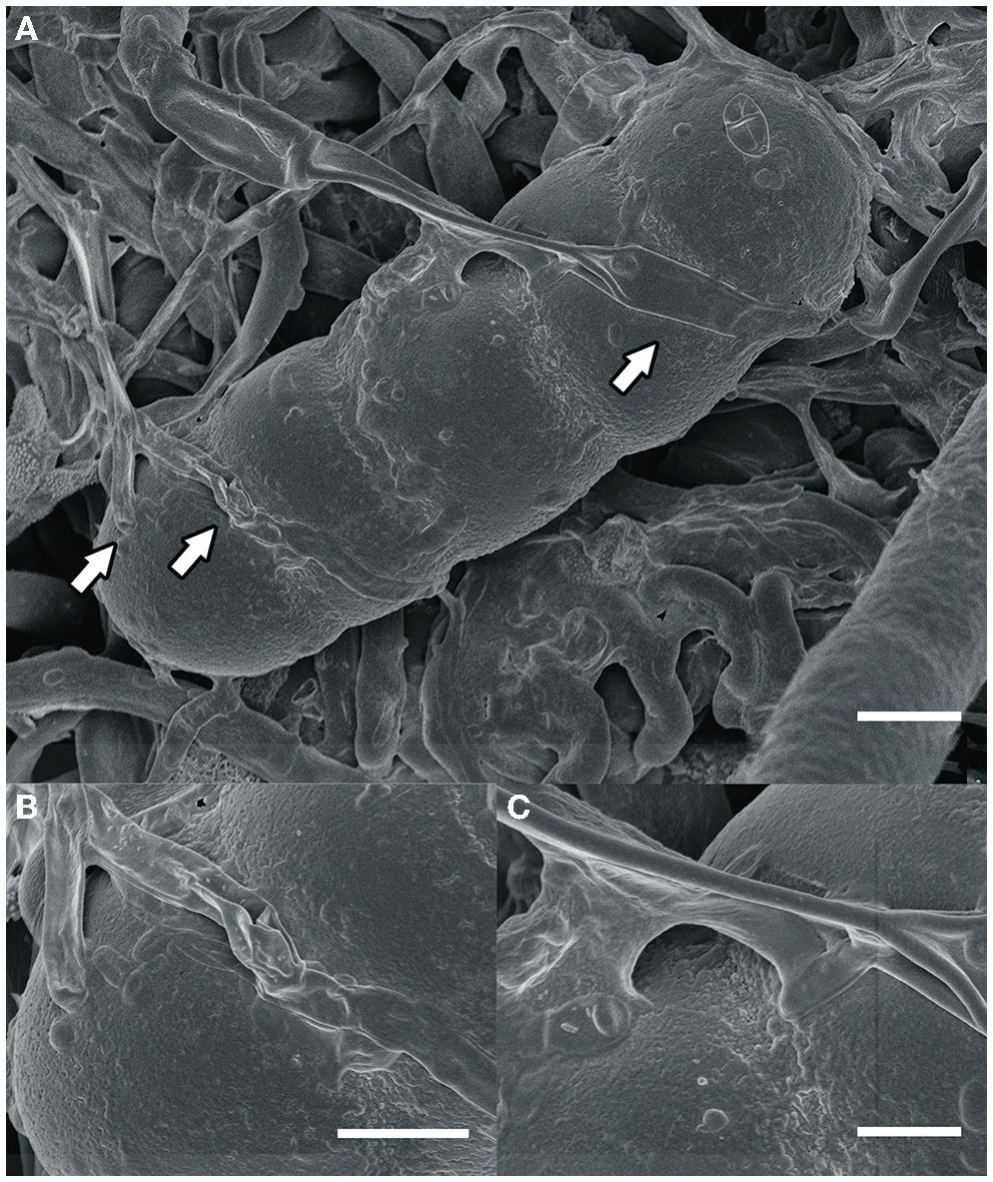
**(A)** An ascospore of *Meliola* sp. and hyphae of the hyperparasitic fungus *Dimerosporiella cephalosporii* (white arrows), as seen by scanning electron microscopy. Bar, 9 μm **(B,C)** close-ups of details indicated by arrows in **(A)**. Bars, **(B)** 3 μm, **(C)** 2 μm.

Most fungi that grow on colonies of Meliolales are obligate biotrophs, as they are found in the field only together with the parasitic host, and there is no history of cultivation on artificial media. Based on morphological and physiological observations only (Jeffries, 1995), nutrients are possibly transferred *via* the interface. In fact, our microscopic observations of material from Panama (Figure 2) indicate that these fungi establish an intimate contact with the hyphae of the host (contact/fusion biotrophs) without the presence of haustoria.

Hyperparasitic interactions are difficult to prove but may be assumed when the parasite causes distinctive morphological or physiological alterations in the host (Jeffries, 1995). In the case of species of Meliolales, hyperparasitic fungi may overgrow their colonies, and prevent the black mildew fungus from producing spores and ascomata (Stevens, 1918; Toro, 1952). Hyperparasites also modify some vegetative structures of Meliolales, such as the density and branching of hyphae, the number, shape or size of hyphopodia, and the presence, number, disposition, size, and shape of setae (Ciferri, 1955). This antagonistic activity and the incapability of hyperparasitic fungi to grow on artificial media strongly suggests that they are obligate parasites (Jeffries, 1995).

### 2.2. Morphological Classification

**Figure 3:**
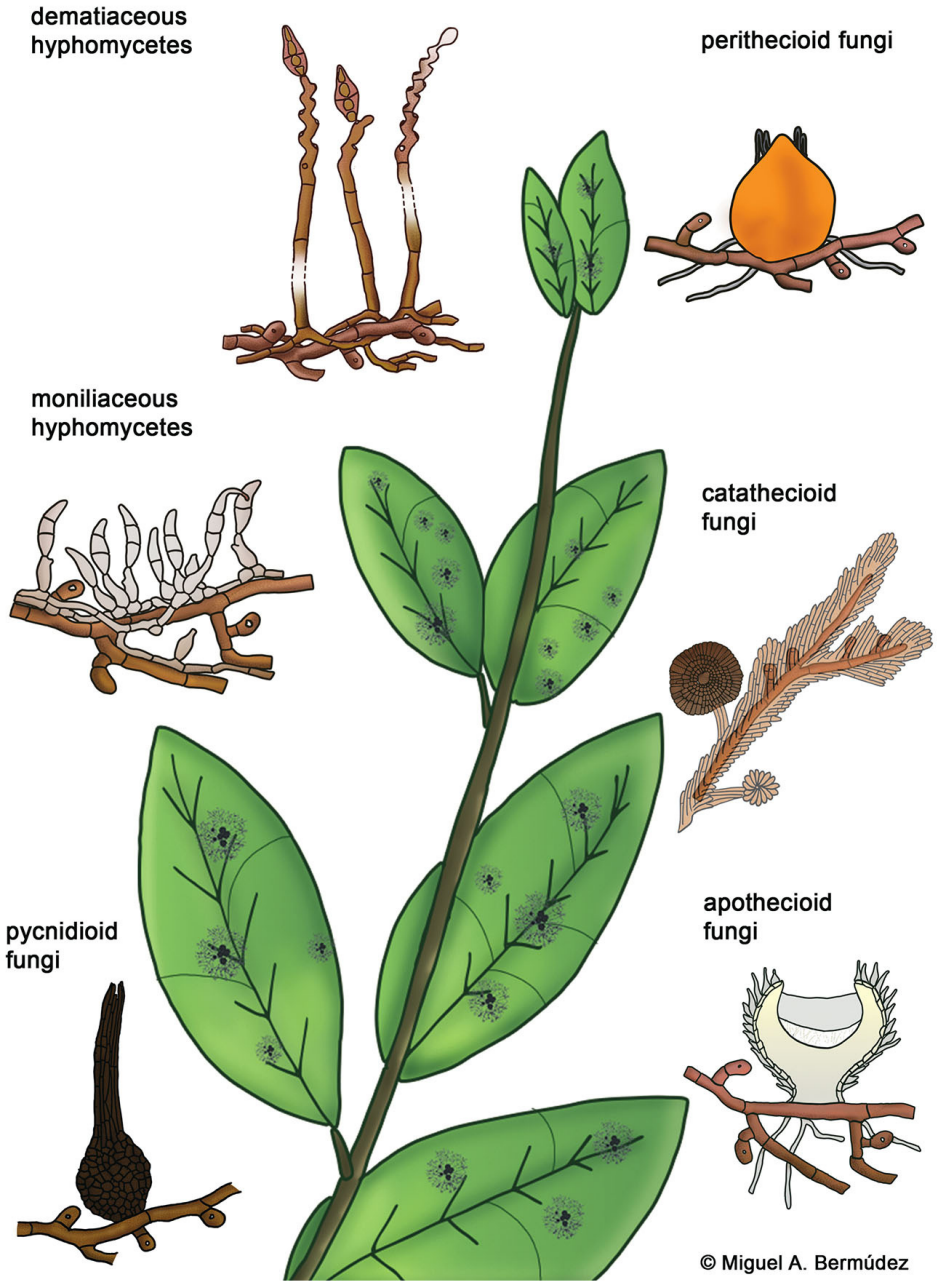
Morphological groups of contact-biotrophic hyperparasitic fungi growing on colonies of Meliolales.

Hyperparasitic fungi form an ecological guild and include organisms from diverse taxonomic groups. Systematic positions of these fungi are mostly unknown and they are poorly represented in sequence databases. Here we use traditional morphological categories to classify 189 species of hyperparasitic fungi that grow on colonies of species of Meliolales (Supplementary Material 1): dematiaceous hyphomycetes, moniliaceous hyphomycetes, pycnidioid, perithecioid, catathecioid, and apothecioid fungi (Figure 3).

#### 2.2.1. Dematiaceous Hyphomycetes (21 Genera, 40 Species)

The artificial group of “dematiaceous” or “dark hyphomycetes” comprises conidial fungi that have heavily melanized, brown-pigmented hyphae and do not form fruiting bodies (Revankar and Sutton, 2010). All genera within this group comprise hyperparasitic species as well as fungi parasitic of other fungi and plants.

**Figure 4:**
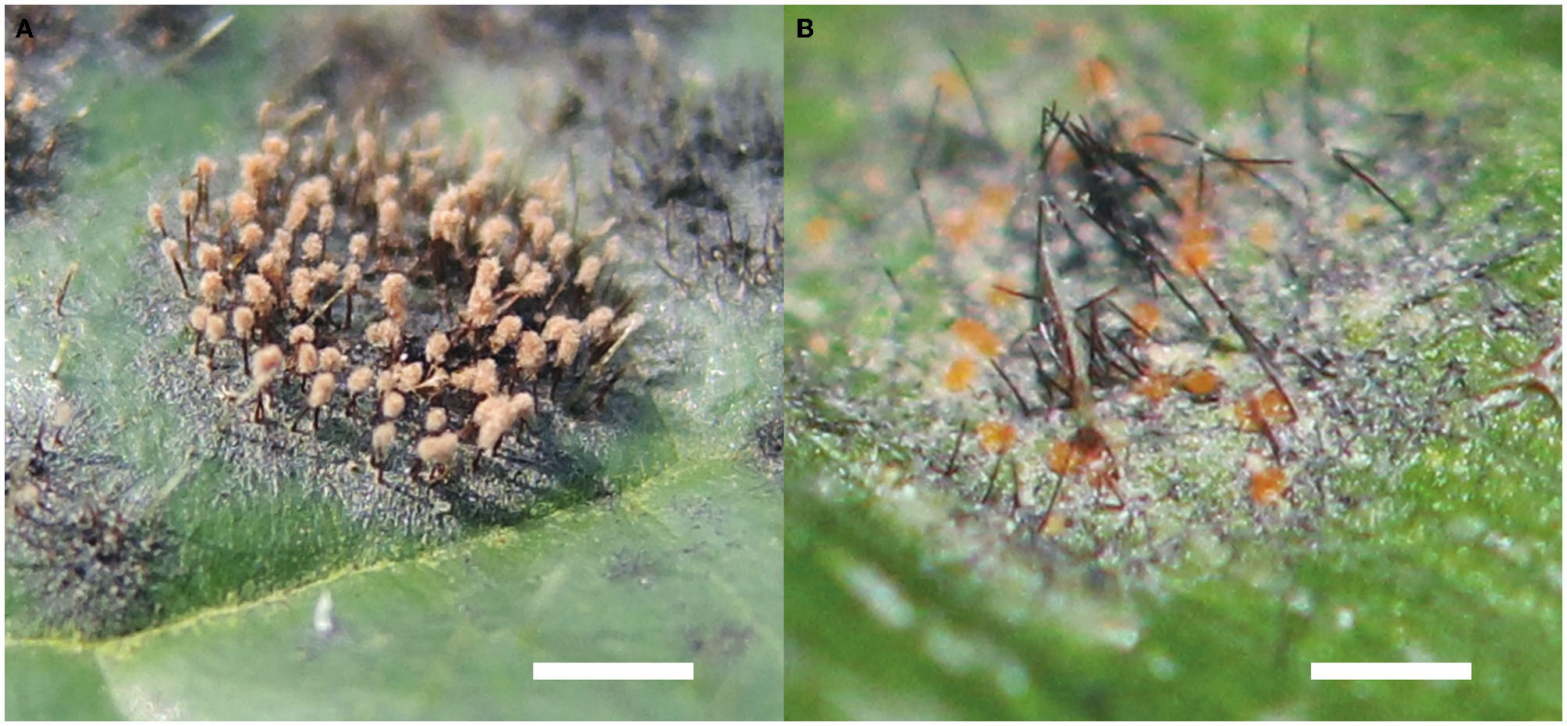
Hyperparasitic fungi of Meliolales. **(A)** Synnemata of *Atractilina parasitica* growing on *Meliola clerodendricola*, on a leaf of *Clerodendrum capitatum*. Bar, 1 mm. **(B)** Orange perithecia of *Dimerosporiella cephalosporii* on black superficial hyphae among setae of *Meliola* sp., on a leaf of *Olyra latifolia*. Bar, 1 mm.

*Atractilina parasitica*, one of the most common hyperparasites of Meliolales, form distinctive straw colored synnemata which are composed of aggregated conidiophores (Figure 4A). This fungus grows almost exclusively on black mildew hosts and has been reported mostly for Africa (Deighton and Pirozynski, 1972). Other common hyperparasitic dematiaceous fungi of black mildews are species of *Helminthosporium* and *Spiropes*. In the past, they were sometimes considered to correspond to conidial stages of species of Meliolales (Ciferri, 1955).

#### 2.2.2. Moniliaceous Hyphomycetes (30 Genera, 52 Species)

Conidial fungi without fruiting bodies and not or only slightly pigmented cells are grouped as moniliaceous hyphomycetes. Some species in this group are only known on black mildews, e.g., *Acremoniula suprameliola, Chionomyces chorleyi, Chionomyces meliolicola, Eriomycopsis biseptata, Trichoconis hamata*, as well as the following four species representing monotypic genera: *Divinia diatricha, Monosporiella meliolicola, Spermatoloncha maticola*, and *Tuberculispora jamaicensis* (Hansford, 1942; Hawksworth, 1981). Other species of hyperparasitic moniliaceous hyphomycetes are more flexible concerning their fungal host range.

#### 2.2.3. Pycnidioid Fungi (5 Genera, 10 Species)

Species of five genera of asexual fungi forming conidia in pycnidia have been reported as parasites of black mildews, namely *Capitorostrum, Chaetophoma, Cicinnobella, Coniothyrium*, and *Naemosphaera* (Stevens, 1918; Petrak, 1950; Hawksworth, 1981). These genera also comprise species parasitic on plants or on other fungi.

#### 2.2.4. Perithecioid Fungi (23 Genera, 68 Species)

Perithecioid hyperparasites develop perithecia containing asci to produce spores. Many genera of this group have been revised by Batista and da Silva (1960), Pirozynski (1977), Rossman (1987), and Rossman et al. (1999). Some examples are the bitunicate ascomycetes of the genera *Paranectriella* and *Puttemansia*, and species with unitunicate asci in *Nematothecium* and *Rizalia*. *Dimerosporiella cephalosporii* (Figure 4B) is one of the most common parasites of *Meliola* spp. in the tropics (Gams et al., 2004). All 11 species of the genus *Melioliphila* are parasites specifically of colonies of black mildews.

#### 2.2.5. Catathecioid Fungi (1 Genus, 17 Species)

Species of the genus *Trichothyrium* are strictly hyperparasitic, and they grow on colonies of Asterinales, Meliolales and other foliicolous species of Ascomycota (Piepenbring, 2015). They are characterized by the presence of catathecia, i.e., flattened perithecia with a well-developed upper and lower peridial wall, and densely packed hyphae that form bands covering hyphae of the host fungus. The delimitation of species in this genus is ambiguous, as only a few morphological characteristics are used, such as the size of ascospores (Wu et al., 2011; Hongsanan et al., 2020).

#### 2.2.6. Apothecioid Fungi (2 Genera, 2 Species)

Two species of fungi with apothecia are known as hyperparasitic fungi on Meliolales. The genus *Unguiculella* comprises mostly saprotrophic species, and *U. meliolicola* is the only species in this genus known to be parasitic on black mildews (Dennis, 1955). The situation for the genus *Calloriopsis* is similar with *C. herpotricha* being the only species hyperparasitic on Meliolales (Sydow and Sydow, 1917, cited as *C. gelatinosa*).

### 2.3. Ecology of Hyperparasitic Interactions

Hyperparasitic fungi may shape the dynamics of the interaction between the plant host and the host fungus, increase the complexity of the food webs and play a significant role in regulating population sizes (Gleason et al., 2014; Sandhu et al., 2021). Hyperparasitic fungi decrease the fitness of the host fungus by inducing hypovirulence and increasing its death rate, eventually clearing the parasitic infection and leading to an uninfected host (Northrup et al., 2021; Sandhu et al., 2021). These effects, to some extent, exert a positive effect on the fitness of host plants and may be used in the context of biocontrol (Kiss, 2001). In the specific case of Meliolales, the population ecology of the host fungus is affected by decreased sporulation (Jeffries, 1995). This limits the dispersal and extension rates of the plant-parasitic fungus. However, Hawksworth (1981) observed that the largest colonies of black mildews are often the richest in hyperparasitic fungi, suggesting that the hyperparasitic fungi may not be really harmful. More in-depth studies on the ecology of these organisms are necessary in order to understand the type of interaction they have with their hosts.

The surface of the setae and of other cells of meliolalean fungi is hydrophilic, thus the colony is easily wetted. This characteristic results in a prolonged state of moisture of the colony and allows the growth of other organisms that use this specific niche. We observed algae, like *Cephaleuros virescens*, yeasts, cyanobacteria, other bacteria and small animals, like mites and tardigrades, in the colonies of black mildews. Metabolites excreted by these organisms and the nitrogen fixed by cyanobacteria may serve as sources of nutrients for Meliolales and may promote the growth of hyperparasites (Piepenbring et al., 2011; Piepenbring, 2015). According to Kiss (2001), a hyperparasitic interaction consists of three trophic levels, but interactions between plants, plant parasites, hyperparasites and these other organisms are certainly more diverse and complex than these three levels indicate.

### 2.4. Analysis of the Species Checklist: Evidencing the Gaps of Knowledge

#### 2.4.1. Species Richness of Hyperparasitic Fungi

To date, no precise number of species of fungi parasitizing black mildew exists. Gams et al. (2004) estimated approximately 75 species of fungi parasitic on black mildews and other leaf-inhabiting fungi. A similar number is found in the species checklist presented by Sun et al. (2019): among 1552 species of fungicolous fungi, i.e., fungi that grow on other fungi that are not necessarily parasitic, 78 species of hyperparasites on Meliolales are reported.

The checklist of hyperparasitic fungi growing on Meliolales presented here is based on primary literature, i.e., scientific publications in international journals with peer review process, and books with ISBN number, as well as secondary literature like review papers, databases, and lists. The publications were found in Google Scholar, Cybertruffle (Minter, 2020), Biodiversity Heritage Library (Gwinn and Rinaldo, 2009), and by references in the analyzed publications. A list with information on type data of species of hyperparasitic fungi on black mildews was obtained from data compiled in Index Fungorum (Kirk, 2019). The checklist (Supplementary Material 1) contains information for records of hyperparasitic fungi growing on Meliolales in an Excel file, including valid scientific names; systematic positions; names of fungal and plant hosts; family of plant hosts; morphological classification; synonyms according to Index Fungorum, MycoBank (Crous et al., 2004), and Zeng et al. (2022); geographic distribution; and references (see Supplementary Material 2). Data analyses were performed with R v4.1.2 (R Core Team, 2022). The package maps v3.4.0 (Becker et al., 2021) was used to draw maps of the different ecoregions, and functions in the package vegan v2.5-7 (Oksanen et al., 2020) were used to build curves of species accumulation with sampling covering, based on the number of records. An R script modified from Piepenbring et al. (2020) was also used for the analyses of the checklist data. Synonyms were no included in the analyses.

**Figure 5:**
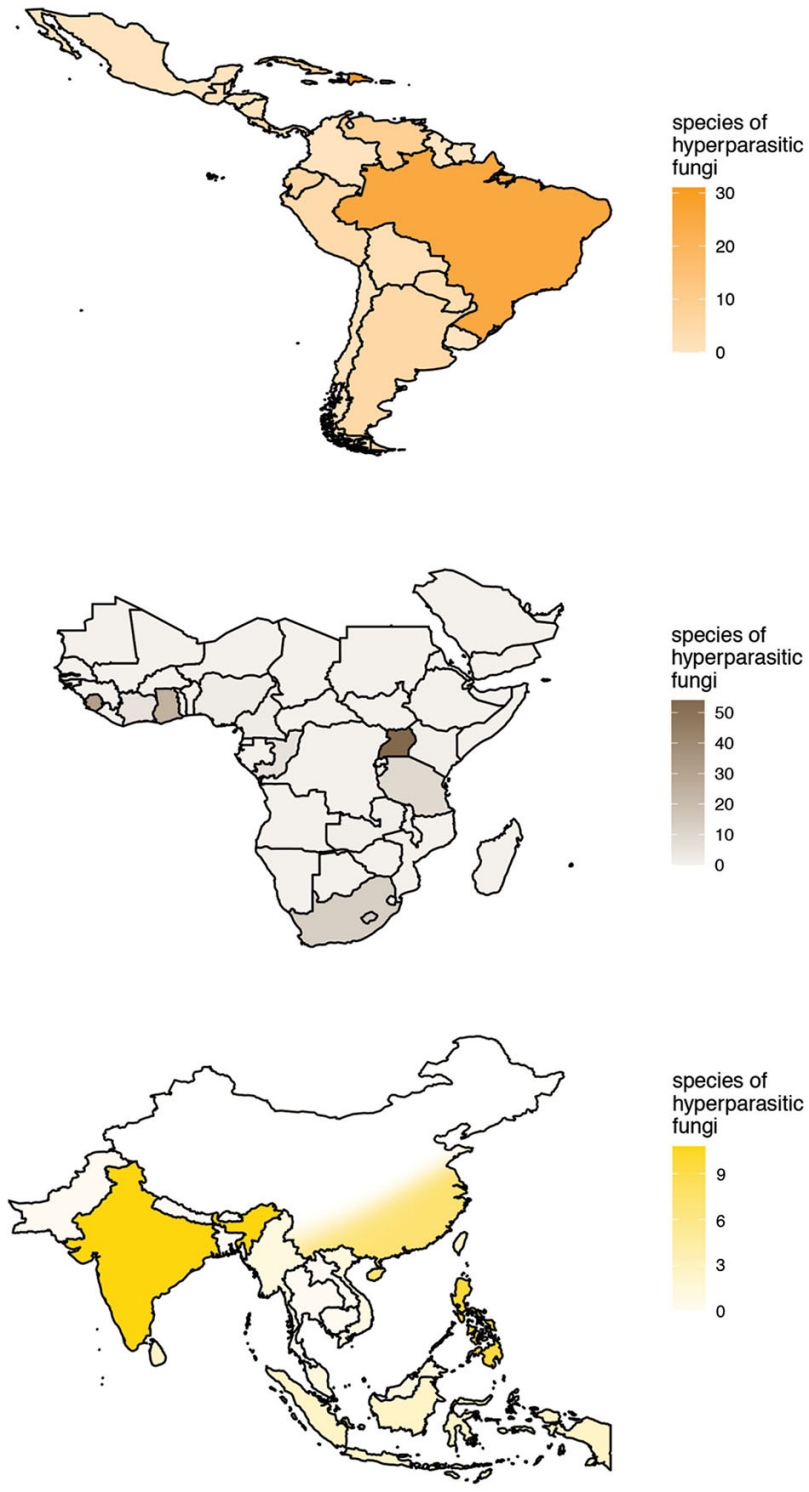
Known species richness and geographic distribution of hyperparasitic fungi on Meliolales in the Neotropics, Afrotropics, and Indomalayan ecoregions according to checklist data. Maps with color intensities relative to the number of hyperparasitic species known per country.

The checklist contains 525 records of hyperparasitic fungi known from all over the world. These refer to 189 species of hyperparasitic fungi growing on colonies of Meliolales, comprised in 82 genera. Thereby, we report more than twice as many hyperparasitic species as cited by other authors up to now. Records were retrieved from 86 publications (Supplementary Material 2). The number of known species of hyperparasitic fungi is maximal in the afrotropics for Uganda (54), followed by Sierra Leone (31) and Ghana (24). In the neotropics, 31 species of hyperparasitic fungi are reported for Puerto Rico, 30 for the Dominican Republic and 25 for Brazil; and in the indomalayan ecoregion, nine and eight species have been reported for India and the Philippines, respectively. The geographic distribution of the species richness known per country is plotted in Figure 5, with color intensities relative to the number of species known per country. Only the most species-rich ecoregions are shown in the graphs.

**Figure 6:**
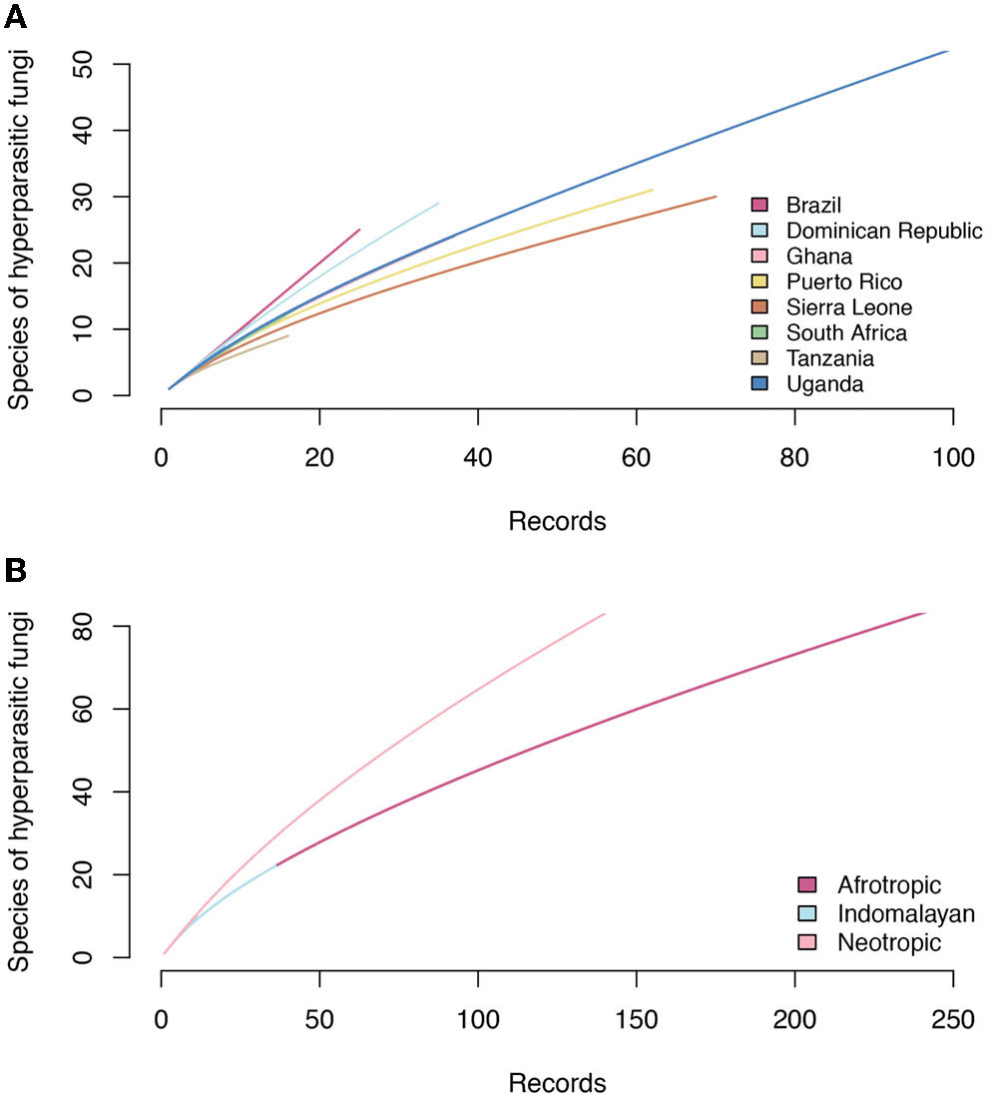
Accumulation curves of hyperparasitic fungi in Meliolales in the Neotropics, Afrotropics, and Indomalayan ecoregions based on **(A)** increasing numbers of publications that were analyzed, and **(B)** on the number of hyperparasitic species known per countries based on increasing numbers of records.

Accumulation curves for hyperparasitic fungal species known for the neotropics, the afrotropics and the Indomalayan region do not reach saturation for any country (Figure 6). Thus, sampling and documentation of the diversity of hyperparasitic fungi in the tropics and subtropics is still incomplete.

The records in the checklist were extracted from literature and adjusted to the checklist concept to the best of our knowledge. Nevertheless, the checklist is still incomplete and some information may not be correct due to the following reasons.

- As the information on hyperparasitic fungi on Meliolales is scattered through literature, it is very likely that further records of hyperparasitic species are hidden in literature.
- Some relevant publications were not available for analysis, as they are hidden in old, local and/or inaccessible journals.
- Identifications of species of hyperparasites and parasites published in literature may not be correct.
- As the species have only been described morphologically, the systematic position of hyperparasites is not resolved and the delimitation of most genera is not well known.

#### 2.4.2. History of Description of Hyperparasitic Fungi

The first scientific investigation of hyperparasitic fungi growing on colonies of Meliolales started in the 1800s with the work of Carlo Spegazzini (Spegazzini, 1889) through an inventory of fungal species in Patagonia, Argentina. The oldest name of a hyperparasitic fungus of Meliolales is *Peziza herpotricha* Berk. (current name: *Calloriopsis herpotricha*), which, however, was not recognized as a hyperparasite by Berkeley (Hooker, 1851). In the following years, only few reports of hyperparasitic fungi are mentioned mainly in publications dealing with individual groups of fungi, or in species inventories (e.g., Patouillard, 1892; Hennings, 1904; Sydow and Sydow, 1917). Some publications center around hyperparasitic fungi on different hosts (primary literature: e.g., Hansford, 1946; Batista et al., 1966; Deighton and Pirozynski, 1972; Pirozynski, 1977; Katumoto, 1987; review papers: Hawksworth, 1981; Gams et al., 2004; Sun et al., 2019), and only a few publications focus specifically on Meliolales and their parasites (Stevens, 1918; Ciferri, 1955; Farr, 1969).

**Figure 7:**
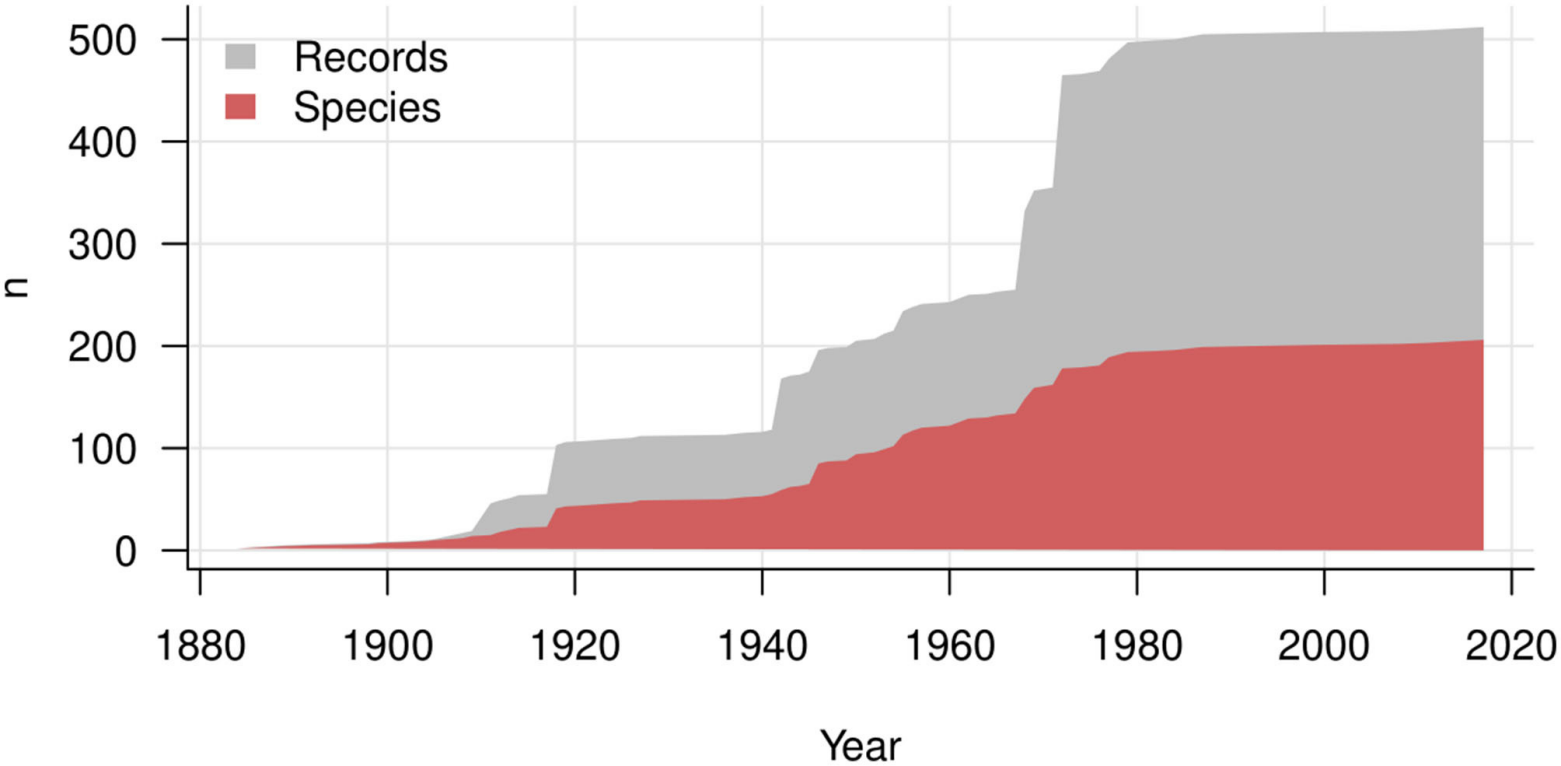
History of description of hyperparasitic fungi on Meliolales over years. The gray area shows the increasing number of records, and the red area the cumulative number of species and infraspecific taxa.

Major contributions are exhibited as jumps in the accumulation lines of records in Figure 7. These contributions include publications by Stevens (1918: 14 species reported for Puerto Rico), Hansford (1946: 17 species reported mostly for Uganda and Ghana), Ellis (1968: 13 species of the genus *Spiropes*) and Deighton and Pirozynski (1972: 16 species reported mostly for Africa). The corresponding jumps are lower than the numbers of records, because many species were reported more than once. As a result, the total number of records has increased much more rapidly than the total number of known species since the 1980s. A plateau of the curves of records and species indicates that hyperparasites on Meliolales were not investigated during the last 20 years, except for one new species combination, *Trichothyrium peristomale*, proposed by Wu et al. (2011). Most current studies on hyperparasitic fungi have focused on their use in biocontrol experiments, which are directed toward reducing the damage caused by a plant pathogen (Day, 2002). Meliolales and their hyperparasites are not aggressive parasites, thus researchers have focused on hyperparasites that cause high mortality of a primary parasite, e.g., hyperparasites on rust fungi.

#### 2.4.3. Systematic Position of Hyperparasitic Species

All 189 taxa in the checklist (Supplementary Material 1) are species of Ascomycota. Among them, a total of 110 species are “incertae sedis” (“uncertain position”) for one or several levels of classification. For 61 species, the systematic position at class level is unknown; for 106 species, the systematic position at order level is unknown, and for 67 species, the systematic position at family level is unknown.

Some conidial forms, especially dematiaceous and moniliaceous hyphomycetes may represent anamorphic stages of certain teleomorphic hyperparasitic fungi that grow on colonies of species of Meliolales. *Dimerosporiella cephalosporii*, for example, is usually found together with an *Acremonium*-like anamorph (Gams et al., 2004). Species of the genus *Isthmospora* are considered as conidial stages of *Trichothyrium* spp. (Ciferri, 1955). Conidia of these hyphomycetes, however, may also be found without perithecia or catathecia. To date, the anamorph-teleomorph connection of many hyperparasitic fungi remains elusive, and it is difficult to determine the precise number of species.

Concepts of genera are based on morphological characteristics and on short Latin descriptions. Fresh collections and DNA sequence data are necessary to establish natural concepts of genera and to elucidate their systematic position. Molecular investigation may also provide evidence on further anamorph-teleomorph connections.

### 2.5. Network Analysis of Host Ranges of Hyperparasitic Fungi

To document and understand the diversity and specificity of species interactions, network theory is frequently used in ecological research. Species are represented as units (nodes) that form interactions (links). This approach serves to visualize species interactions (Pocock et al., 2016) or to characterize the structure of ecological communities (Dormann et al., 2009). Most studies on tritrophic parasitic networks (in the wider sense) were conducted on phytophagous insects and their insect parasites or parasitoids that infect them (Derocles et al., 2018; de Araujo and Maia, 2021; Kawatsu et al., 2021). Network theory has not yet been applied to fungal hyperparasitic-host fungus interactions.

**Figure 8:**
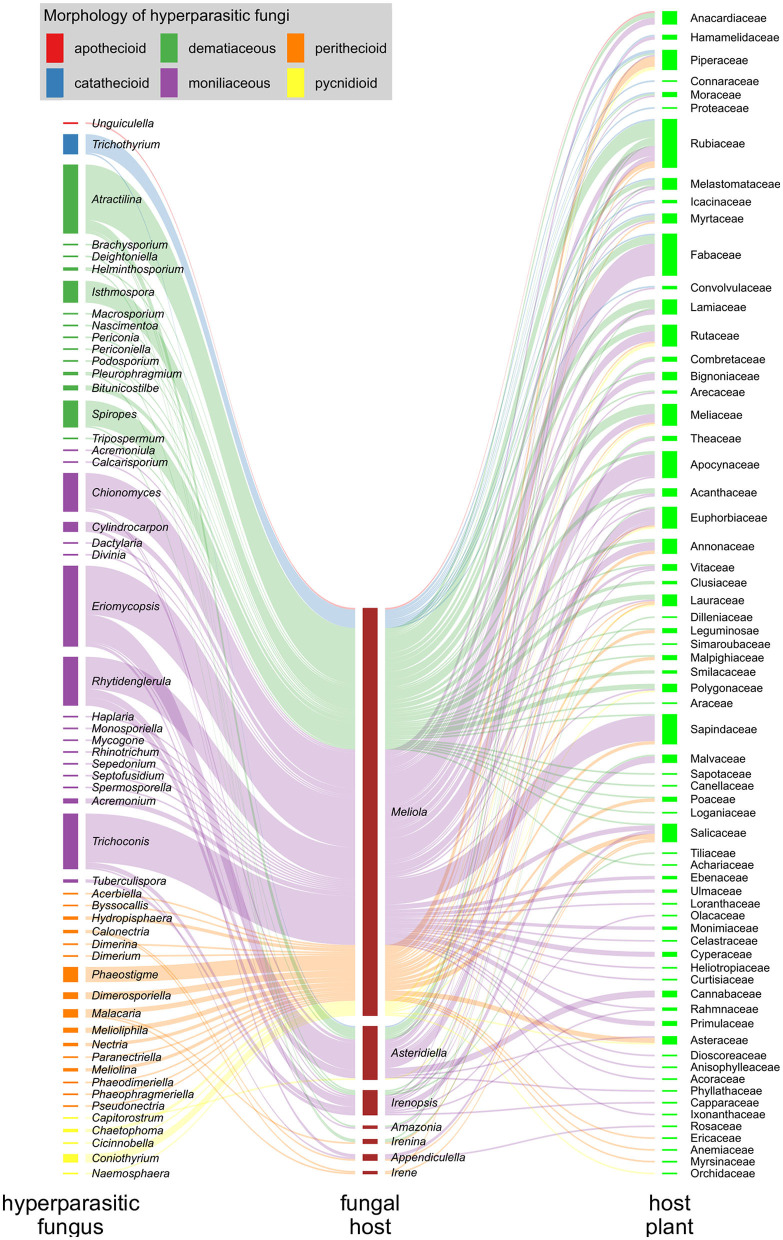
Hyperparasitic fungi-Meliolales-Host plant tritrophic interaction network. Nodes (colored rectangles) represent genera (hyperparasitic fungi, host fungus) or families (host plants), and links (lines) represent species interactions. The width of the nodes and links corresponds to the frequency of records of species interactions. Hyperparasitic fungal species are represented as genera on the left side of the network graph, with colors referring to the morphological group to which they belong; Meliolales species nodes are dark red, and nodes of host plant families are green.

In Figure 8, we illustrate the interactions of hyperparasitic fungi infecting species of Meliolales, which are themselves parasitic on plants (Supplementary Material 1), in a network. Fungal hyperparasites and their fungal hosts are grouped by genus, and their plant hosts by family. Hyperparasitic interactions with fungal and plant hosts not identified to genus and family level respectively were excluded. The network was visualized using the packages ggforce v0.3.3 (Pedersen, 2021) and ggplot2 v3.3.5 (Wickham, 2016) in R v4.1.2 (R Core Team, 2022). Colors were used to highlight morphological groups of hyperparasitic fungi.

The graph is based on 300 records of species of hyperparasitic fungi that were found on different genera of Meliolales. Moniliaceous hyphomycetes were observed most frequently, followed by dematiaceous hyphomycetes and perithecioid fungi (Figure 8). The abundance of genera of Meliolales reflects the abundance and known species richness of genera of Meliolales, with *Meliola* being be far the most frequent and species rich genus. The abundance of plant host families reflects known host preferences of species of Meliolales among species of angiosperms, with Apocynaceae, Euphorbiaceae, Fabaceae, Rubiaceae, and Sapindaceae, presenting an elevated number of species of Meliolales.

The host range of most genera of hyperparasitic fungi includes several species of one or several genera of Meliolales, i.e., hyperparasitic fungi are generalists concerning their hosts among Meliolales. The network graph shows a notorious preference of most species of hyperparasitic fungi for species of the genus *Meliola*, independently of the generic position and the morphological group of the hyperparasitic fungus. As a genus with diverse and abundant host species, the chances of *Meliola* spp. being colonized by hyperparasitic fungi are higher than for species of other genera of Meliolales. According to Vazquez et al. (2005), for host-parasite systems, the more abundant host taxa tend to have a higher diversity of parasites and to have a higher representation of specialist parasites.

The association between hyperparasitic fungi and host plants is diverse and aleatory, and no correlation between both groups is observed. Host plant diversity does not depend on the hyperparasites but on the host fungi (Meliolales), that are known to be host specific at the level of species, genera, or families (Jayawardena et al., 2020).

Concerning the conclusions drawn from this analysis, several important aspects need to be considered.

- For 31 species of hyperparasitic fungi, associations are represented only by a single specimen. In this case, a single connection is shown in the graph, suggesting that these species of hyperparasitic fungi are highly specific. This is most likely not the case, when sampling efforts are increased.
- In addition to the susceptibility of the host fungi, numerous further factors are important for the occurrence of parasite-hyperparasite interactions, especially environmental conditions (Bryner and Rigling, 2011; Kohl et al., 2019) and the availability of inoculum. There are no data available to further discuss these aspects.
- Genera of Meliolales are based on morphological characteristics and preliminary sequence data shows that new circumscriptions and placement of genera will be required (Mibey and Hawksworth, 1997; Marasinghe et al., 2020; Jayawardena et al., 2021; Zeng et al., 2022). The non-specificity between groups of hyperparasitic fungi and genera of Meliolales may be a consequence of the fact that meliolalean genera are artificial. We do not expect, however, to see host specificity even with natural genera.

Beyond data presented in this network analysis, it is important to mention that not all species of hyperparasitic fungi are restricted to meliolalean hosts. *Eriomycopsis flagellata* , for example, parasitizes colonies of *Asteridiella* and *Meliola* (Meliolales), *Asterina* (Asterinales), and *Balladyna* (Balladynaceae). Nevertheless, literature research and our sampling experience indicate that most species of hyperparasitic fungi are restricted to meliolalean hosts.

In the case of species of hyperparasitic fungi for which several records are available, broad host spectra are observed. For *Eriomycopsis bomplandi*, for example, 27 records are available, referring to 22 different host species. Apparently, hyperparasitic fungi are generalists not only at the genus level, but also at the species level of the host fungus.

Building multitrophic ecological networks is a difficult task, especially in poorly studied and highly diverse systems (Derocles et al., 2018), as is the case for hyperparasitic fungi and black mildews. Sampling efforts need to be increased and data from more countries and host fungi should be included to strengthen future analyses of these species' interactions (Cazabonne et al., 2022).

### 2.6. Problems Related to Molecular Sequencing of Hyperparasitic Fungi

To date, no sequencing data are available for any fungal species hyperparasitic on Meliolales. Here we present some reasons that might have prevented the development of methods for molecular studies of these organisms:

**a. Strong melanization**. Melanin is a ubiquitous compound that is present in many fungal cell walls with varying quantities depending on the species (Revankar and Sutton, 2010). For example, species of the genus *Spiropes*, common hyperparasites of Meliolales, have a tough surface layer of melanin in their cell walls. This inert polymer is insoluble at cold temperatures and impermeable to boiling and organic solvents. Melanin is also highly resistant to UV light, acids, and enzymatic digestion (Karakousis et al., 2006). According to Eckhart et al. (2000), melanin is also a potent inhibitor of thermostable DNA polymerase, and the inhibitory effect is conferred by a direct and reversible polymerase-melanin interaction.

**b. Biomass**. The reproductive structures of hyperparasitic microfungi, when present, are less than 1 mm in size and are present in limited quantities. This makes the extraction procedures difficult, as many DNA extraction methods depend on adequate biomass of the organism.

**c. Mixed-infections**. Isolating DNA from only one specific hyperparasite without contamination by other organisms remains challenging. DNA sequences resulting from these samples might be attributed to the wrong species.

**d. The lack of DNA sequences for comparison**. As there are no DNA sequences available for any species of mycoparasites of Meliolales, no reference sequences exist. Apparently most hyperparasitic fungi of black mildews are obligate biotrophs and cannot be grown separate from their hosts. The hosts themselves are also biotrophic parasites making it challenging to isolate and sequence the hyperparasites.

**e. No single method**. Hyperparasitic fungi have different morphologies and belong to diverse systematic relationships. Therefore, the molecular methods to study them may vary depending on each group.

The development of methods to study the DNA of hyperparasitic microfungi is a necessary task in order to better understand the diversity and evolution of this guild of fungi.

## 3. Discussion

By the present contribution, information on species of fungi hyperparasitic on Meliolales is compiled in a checklist for the first time. Checklists on species diversity are essential sources of information for the characterization of biodiversity in any given area. These lists help to understand the present state of knowledge of fungi in the area and provide information on the ecology, taxonomy and biogeography of fungi, especially of undersampled taxonomic and ecological groups (Piepenbring et al., 2020). The determination of fungi in the tropics is a great challenge due to the lack of monographs, reference specimens and expertise (Piepenbring et al., 2018). Moreover, there is no detailed treatment of biotrophic plant pathogens and their parasites (Gams et al., 2004) as most publications deal with individual groups of fungi.

The huge diversity of reproductive structures presented by hyperparasitic fungi on Meliolales indicates the polyphyletic nature of this ecological group. Colonies of Meliolales were “discovered” repeatedly during evolution by fungi belonging to different systematic groups.

In the context of the present study, a tritrophic network analysis of fungi hyperparasitic on plant-parasitic fungi is presented for the first time. Hyperparasitic fungi are generalists concerning genera of Meliolales. This can be explained by the fact that they are contact parasites and do not penetrate into host cells. However, most species of hyperparasitic fungi are specific to Meliolales, probably due to the specific growth conditions provided by the meliolalean colonies, i.e., moisture and metabolites of associated microorganisms.

As meliolalean fungi and their hyperparasites are not aggressive parasites, they are not in the focus of applied mycological research. However, we need further morphological, molecular and ecological studies on these fungi in order to understand their diversity, evolution and biology.

## Author Contributions

MB-C compiled and analyzed the data and wrote the first draft. AC-L performed the network analysis. MB-C, AC-L, and MP contributed to writing and editing the manuscript. All authors contributed to the article and approved the submitted version.

## Funding

MB-C acknowledges support from the German Academic Exchange Service (DAAD), within the framework of the scholarship program for doctoral studies in Germany (Ref. no.: 91726217).

## Conflict of Interest

The authors declare that the research was conducted in the absence of any commercial or financial relationships that could be construed as a potential conflict of interest.

## Publisher's Note

All claims expressed in this article are solely those of the authors and do not necessarily represent those of their affiliated organizations, or those of the publisher, the editors and the reviewers. Any product that may be evaluated in this article, or claim that may be made by its manufacturer, is not guaranteed or endorsed by the publisher.

## Supplementary Material

Supplementary Material 1 Checklist of hyperparasitic fungi on Meliolales known for the tropics and subtropics based on literature.

Supplementary Material 2 References to literature containing records of hyperparasitic fungi on Meliolales for tropical and subtropical countries and cited in Supplementary Material 1.
