# Supplementary material for: Hyperparasitic Fungi on Black Mildews (Meliolales, Ascomycota): Hidden Fungal Diversity in the Tropics
Source: Front Fungal Biol. 2022 May 24;3:885279. doi: 10.3389/ffunb.2022.885279 (PMC10512288; doi:10.3389/ffunb.2022.885279)
Supplement: Supplementary Material 2 — References to literature containing records of hyperparasitic fungi on Meliolales for tropical and subtropical countries and cited in Supplementary Material 1. [file Data_Sheet_2.DOCX]

**Supplementary material 2**

List of references used in the species checklist of species of fungi hyperparasitic on Meliolales

1. Batista, A.C. (1962). Contribuição do estudo dos fungos hiperparasitas. Publicações Inst. Micol. Recife 211, 1-51.
2. Batista, A.C, and Bezerra, J.L. (1964). Polystomellaceae: novas entidades Brasileiras. Port. Acta. Biol. Sér. B 7, 361-382.
3. Batista, A. C., and da Silva, J.N. (1953). Dois novos fungos imperfeitos. Anais IV Congr. Soc. bot. Brasil, 144-150.
4. Batista, A. C., and da Silva, M.H. (1957). Alguns *Trichothyria* do Brasil e das Filipinas. 15, Anais. Soc. Biol. Pernambuco 467-478.
5. Batista, A. C., and da Silva, M.H. (1957). Um grupo de fungos hiperparasitas. Revta. Biol. Lisb. 1, 140-156.
6. Batista, A. C., and da Silva, M.H. (1960). Algumas espécies de *Dimerina*, *Dimerium*, e *Phaeodimeriella*, assinaladas como hiperparasitas. Publicações Inst. Micol. Recife 1, 337-347.
7. Batista, A.C., and Peres, G.E.P. (1960). Stilbaceae – duas novas espécies. Publicações Inst. Micol. Recife 266, 1-11.
8. Batista, A.C., and Peres, G.E.P. (1965). Novos Deuteromycetes da micogeografia intercontinental. Mycopath. Mycol. appl. 25, 162-172.
9. Batista, A.C., Bezerra, J.L., da Silva, M.H., and Garnier, R. (1966). Alguns hieperparasitas de Meliolaceae e outros Ascomycetes. Atas. Inst. Micol. Univ. Recife 3, 10-30.
10. Batista, A.C., da Silva, M.H., and Bezerra, J.L. (1965). *Brachysporium minutum* n.sp. e outros Dematiaceae fragmospóricos. Publicações Inst. Micol. Recife 446, 1-19.
11. Chevaugeon, J. (1956). Les maladies cryptogamiques du manioc en Afrique Occidentale. Enciclopédie Mycologique 28. France.
12. Ciferri, R. (1938). Mycoflora Domingensis exsiccata. Annls. Mycol. 36, 198-245.
13. Ciferri, R. (1951). Schedae mycologicae. I-IX. Mycopath. Mycol. appl. 6, 19-27.
14. Ciferri, R. (1954). Schedae mycologicae. XII-XXXIV. Sydowia 8, 245-279.
15. Ciferri, R. (1955). Observations on Meliolicolous Hyphales from Santo Domingo. Sydowia 9, 296-335.
16. Ciferri, R. (1962). Chedae mycologicae. XXXV-XCVIII. Atti. Ist. bot. Univ. Lab. Crittog. Pavia 19, 85-139.
17. Ciferri, R., and Batista, A. C. (1956). A new genus of phaeophragmeous Dematiaceae with pseudoendogenous conidia. Publicações Inst. Micol. Recife 44, 1-5.
18. Deighton, F.C. (1936). List of Fungi collected in Sierra Leone. Kew Bull. 7, 424-433. Stable URL: https://www.jstor.org/stable/4111839
19. Deighton, F.C. (1969). Microfungi. IV: some hyperparasitic hyphomycetes, and a note on *Cercosporella uredinophila* Sacc. Myc. Papers 118, 1-41.
20. Deighton, F.C., and Pirozynski K.A. (1972). Microfungi. V. More hyperparasitic hyphomycetes. Myc. Papers 128.
21. Dennis, R.W.G. (1955). Fungi from Sierra Leone: Pezizales and Helotiales. Kew Bull. 3, 363-368.
22. Doidge, E.M. (1924). South African Ascomycetes in the National Herbarium. Part III. Bothalia 1, 195-221.
23. Dubey, R., and Pandey, A.D. (2019). Statistical analysis of foliicolous fungal biodiversity of Konkan region, Maharashtra, India: A novel approach. Plant Pathology & Quarantine 9, 77-115.
24. Ellis, M.B. (1957). Some species of *Deightnoniella*. Myc. Papers 66, 1-12.
25. Ellis, M.B. (1967). Dematiaceous Hyphomycetes. VIII. *Periconiella, Trichodochium* etc. Myc. Papers 111, 1-46.
26. Ellis, M.B. (1968). Dematiaceous hyphomycetes. IX. *Spiropes* and *Pleurophragmium*. Myc. Papers 114, 1-44.
27. Farr, M. L. (1969). Some “black mildew”, “sooty mold”, and “fly speck” fungi and their hyperparasites from Dominica. Can. J. Botany 47, 369-381.
28. Gams, (1971). *Cephalosporium*-artige Schimmelpilze (Hyphomycetes), i-x. Stuttgart: Gustav Fischer Verlag.
29. Guyot, A.L. (1949). Contribution á l’étude des cryptogames parasites de la France septentrionale. Rev. Mycologie 14, 61-84.
30. Hansford, C.G. (1941). Contributions towards the fungus flora of Uganda. – III. Some Uganda ascomycetes. Proc. Linn. Soc. 153, 4-52.
31. Hansford, C.G. (1942). The genus *Eriomycopsis* Speg. Bothalia 4, 464-473.
32. Hansford, C.G. (1943). Contributions towards the fungus flora of Uganda. – V. Fungi Imperfecti. Proc. Linn. Soc. 155, 34-67.
33. Hansford, C.G. (1944). Contributions towards the fungus flora of Uganda. – VI. New records. Proc. Linn. Soc. 156, 102-124.
34. Hansford, C.G. (1945). Contributions towards the fungus flora of Uganda. – VII. New records and revisions. Proc. Linn. Soc. 157, 20-41.
35. Hansford, C.G. (1946). The Foliicolous Ascomycetes, their parasites and Associated Fungi. Especially as illustrated by Uganda Specimens. Myc. Papers 15, 1-240.
36. Hansford, C.G. (1947a). New or interesting tropical fungi. I. Proc. Linn. Soc. 158, 28-50.
37. Hansford, C.G. (1947b). New tropical fungi. Proc. Linn. Soc. 159, 21-42.
38. Hennings, P. (1904). Fungi amazonici a cl. E. Ule collecti I-III. Hedwigia 43, 242-273.
39. Holubová-Jechová, V. (1982). Lignicolous Hyphomycetes from Czechoslovakia. 6. *Spadicoides* and *Diplococcium*. Folia Geobot. Phytotax. 17, 295-327.
40. Holubová-Jechová, V., and Mercado Sierra, Á. (1984). Studies on Hyphomycetes from Cuba II. Hyphomycetes from the Isla de la Jueventud. Česká Mykol. 38, 96-120.
41. Hughes, S.J. (1953). Fungi from the Gold Coast. II. Myc. Papers 50, 1-104.
42. Katumoto, K. (1987). Five hyperparasitic hyphomycetes from Japan. Bull. Faculty of Agriculture, Yamaguchi University 35, 107-119.
43. Mena-Portales, J., Delgado-Rodríguez, G., Hernández-Gutiérrez, A., González-Fraginals, G., and Mercado-Sierra, Á. (2017). Hyphomycetes from Sierra de Cubitas, Cuba. Acta bot. cuba 216, 17-30.
44. Pande, A. (2008). Ascomycetes of Peninsular India. Jodhpur, India: Scientific Publishers.
45. Patouillard, N. (1892). Champignons nouveaux extra-europeens. Pl. VII. Bull. Soc. Mycol. Fr. 8, 46-56.
46. Patouillard, N.T. (1898). Champignons nouveaux ou peu connus. Bull. Soc. Mycol. Fr. 14, 149-156.
47. Petch, T. (1937). Naturalist 35, 282. (title not found)
48. Petrak, F. (1924). Mykologische Notizen. VII. Annls. Mycol. 22, 1-182.
49. Petrak, F. (1940). Beiträge zur Pilzflora der Umgebung von Wien. Annls. Mycol. 38, 339-386.
50. Petrak, F. (1950). Beiträge zur Pilzflora von Ekuador. Sydowia 4, 450-587.
51. Pietschmann, (1919).
52. Pirozynski, K.A. (1974). *Meliolina mollis* and two hyperparasites in India. Kavaka 2, 33-41.
53. Pirozynski, K.A. (1977). Notes on Hyperparasitic Sphaeriales, Hypocreales and ‘Hypocreoid Dothideales’. Kew Bull. 31, 595-610. Stable URL: https://www.jstor.org/stable/4119409
54. Raciborski, M. (1909). Parasitische und epiphytische Pilze Java’s. Bull. Int. Acad. Sci. Lett. Cracovie, Cl. Sci. Math. Nat. Sér. B., sci. nat. 3, 346-393.
55. Rossman, A. Y. (1979). A preliminary account of the taxa described in *Calonectria*. Mycotaxon 8, 485-558.
56. Rossman, A.Y. (1987). The Tubeufiaceae and similar Loculoascomycetes. Myc. Papers 157.
57. Rossman, A.Y., Samuels, G.J., Rogerson, C.T., and Lowen, R. (1999). Genera of Bionectriaceae, Hypocreaceae and Nectriaceae (Hypocreales, Ascomycetes). Stud. Mycol. 42.
58. Saccardo, P.A. (1884). Sylloge fungorum omnium hucusque cognitorum. Digessit P.A. Saccardo; Vol. III. Sphæropsidae et. Melaconieæ. Italy.
59. Saccardo, P.A., and Saccardo, D. (1905). Sylloge fungorum omnium hucusque cognitorum. Digessit P.A. Saccardo; Vol. XVII. Supplementum universale Pars VI. Hymenomycetae-Laboulbeniomycetae. Italy.
60. Saccardo, P.A., and Trotter, A. (1913). Sylloge fungorum omnium hucusque cognitorum. Digessit P.A. Saccardo; Vol. XXII. Supplementum universale Pars IX. Ascomycetae-Deuteromycetae. Italy.
61. Samson, R.A., Gams, W., and Evans, H.C. (1979). *Pleurodesmospora*, a new genus for the entomogenous hyphomycete *Gonatorrhodiella coccorum*. Persoonia 11, 65-79.
62. Sathe, A.V., and Vaidya, J.G. (1976). *Nematothecium hansfordii* sp. nov. – a new generic record to India. Curr. Sci. India 45, 145-146.
63. Seaver, F.J., and Chardón, C.E. (1926). Botany of Porto Rico and the Virgin Islands. Mycology. Sciènt. Surv. P. Rico 8.
64. Spegazzini, C. (1889). Fungi Patagonici. Boln. Acad. nac. Cienc. Córdoba 11.
65. Spegazzini, C. (1908). Hongos de la yerba mate. Anal. Mus. Nac. B. Aires 17, 111-141.
66. Spegazzini, C. (1910). Mycetes Argentinenses. Series V. Anal. Mus. nac. B. Aires 13, 329-467.
67. Spegazzini, C. (1918). Notas micológicas. Physis. Rev. Soc. Arg. Cienc. Nat. 4, 281-295.
68. Spegazzini, C. (1924). Algunos honguitos Portoriqueños. Boln. Acad. Nac. Cienc. Córdoba 26, 335-368.
69. Stevens, F.L. (1918). Some meliolicolous parasites and commensals from Porto Rico. Bot. Gaz. 65, 227-250.
70. Stevens, F.L., and Dalbey, N.E. (1919). New or noteworthy Puerto Rican fungi. Mycologia 11, 4-9.
71. Sydow, H. (1899). Beiträge zur Kenntnis der Pilzflora der Mark Brandenburg. II. Hedwigia Beiblatt 38, 134-140.
72. Sydow, H. (1927). Fungi in itinere Costaricensi collecti. Pars tertia. Annls. Mycol. 25, 1-160.
73. Sydow, H., and Sydow, P. (1912). Fungi from the island of Palawan. Leafl. of Philipp. Bot. 5, 1533-1547.
74. Sydow, H., and Sydow, P. (1913). Enumeration of Philippine fungi, with notes and descriptions of new species. II. Philipp. J. Sci. C. Bot. 8, 475-508.
75. Sydow, H., and Sydow, P. (1917). Beitrag zur Kenntnis der Pilzflora der Philippinen-Inseln. Annls. Mycol. 15, 165-268.
76. Theissen, F. (1912). Zur Revision der Gattung *Dimerosporium*. Beih. bot. Zbl. 29, 45-73.
77. Theissen, F. (1914). Die Trichothyriaceen. Beih. Bot. Zbl. 32, 1-16.
78. Theissen, F., and Sydow, H. (1918). Synoptische Tafeln. Annls. Mycol.15, 389-491.
79. Tim, S.K.-M. (1971). The morphology and development of *Trichothyrium asterophorum*. Bot. Gaz. 132, 318-326.
80. Toro, R.A. (1952). A Study of the Tropical American Black-mildews. J. Agric. Univ. Puerto Rico 36, 24-87.
81. Viégas, A.P. (1946). Alguns fungos do Brasil. XIII. Hifomicetos. Bragantia 6, 353-442.
82. von Arx, J.A., and Müller, E. (1962). Die Gattungen der didymosporen Pyrenomyceten. Beitr. Kryptfl. Schweiz 11, 1-922.
83. von Höhnel, F.X.R (1908). Fragmente zur Mykologie (VIII. Mitteilung, Nr. 407 bis 467). Sber. Akad. Wiss. Wien, Math.-naturw. Kl. 118, 1461-1552.
84. Wu, H.X., Schoch, C.L., Boonmee, S., Bahkali, A.H., Chomnunti, P., and Hyde, K.D. (2011). A reappraisal of Mycrothyriaceae. Fungal Divers. 51, 189-248. doi: https://doi.org/10.1007/s13225-011-0143-8
85. Zhao, G., Wu, Y., and Li, N. (1996). Six fungi species of hyperparasite on Meliolaceae. Journal of Beijing Forestry University 1, 99-103.
86. Zimmermann, A. (1902). Zentbl. Bakt. ParasitKde Abt. I 8, 321. (titlte not found)
